# Editor's Letter-Box

**Published:** 1888-10-20

**Authors:** 


### Editor's Letter-Box.

[Our Correspondents are reminded that prolixity is a great bar to publication, and that brevity of style and conciseness of statement greatly facilitate early insertion.]

Will you kindly inform me of the best "Glossary" of medical terms used in midwifery, which will also explain doctors' prescriptions? A Student.

["A Student" had better consult a medical publisher.—Ed. *T. H.*]

